# Supplementary material for: Overview of the Saccharomyces cerevisiae population structure through the lens of 3,034 genomes
Source: G3 (Bethesda). 2024 Nov 19;14(12):jkae245. doi: 10.1093/g3journal/jkae245 (PMC11631439; doi:10.1093/g3journal/jkae245)
Supplement: jkae245_Supplementary_Data [file jkae245_supplementary_data.zip › Figure_S5_G3-2024-405400.pdf]

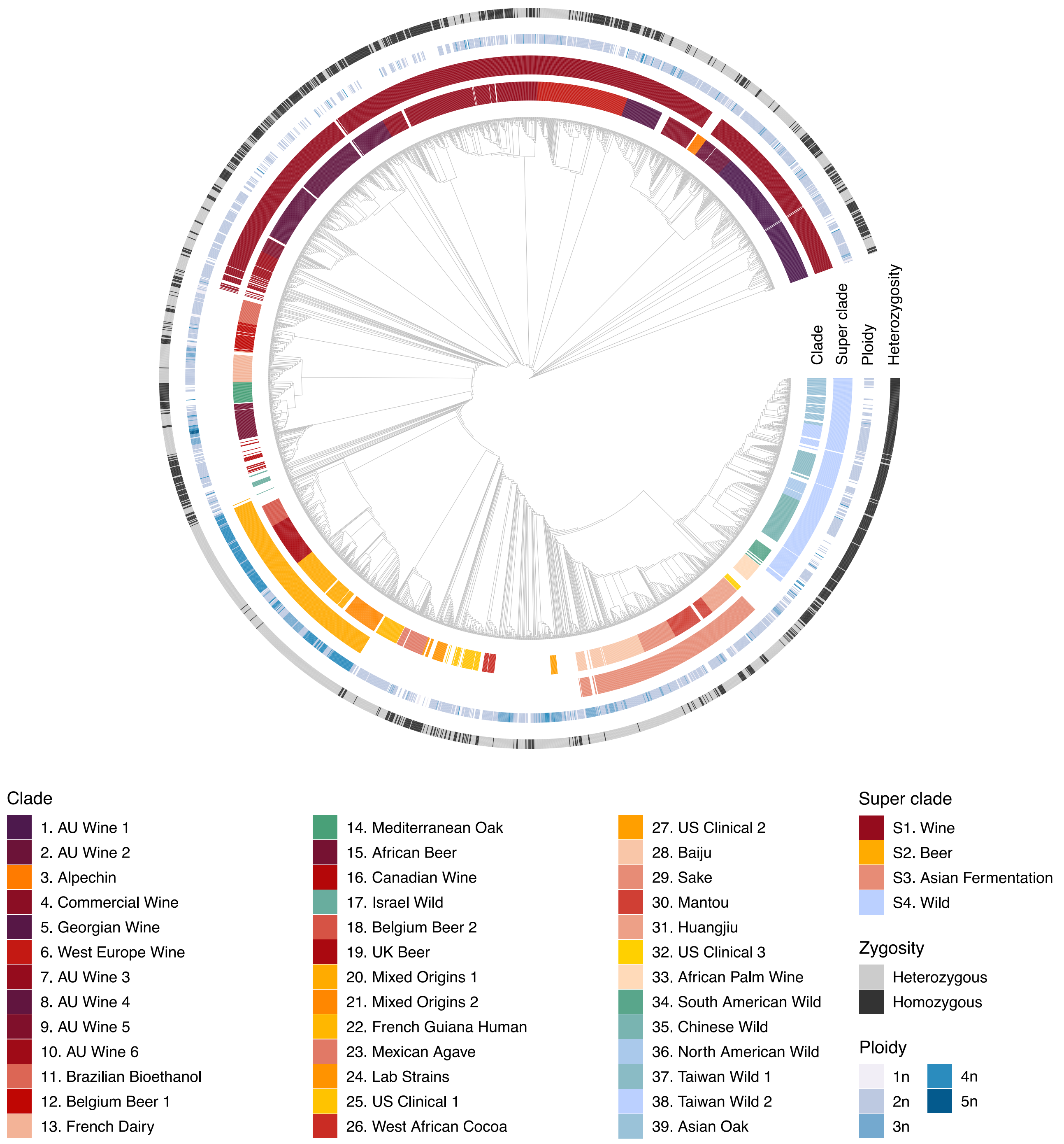

Clade
Super clade
Ploidy
Heterozygosity
Clade
1. AU Wine 1
2. AU Wine 2
3. Alpechin
4. Commercial Wine
5. Georgian Wine
6. West Europe Wine
7. AU Wine 3
8. AU Wine 4
9. AU Wine 5
10. AU Wine 6
11. Brazilian Bioethanol
12. Belgium Beer 1
13. French Dairy
14. Mediterranean Oak
15. African Beer
16. Canadian Wine
17. Israel Wild
18. Belgium Beer 2
19. UK Beer
20. Mixed Origins 1
21. Mixed Origins 2
22. French Guiana Human
23. Mexican Agave
24. Lab Strains
25. US Clinical 1
26. West African Cocoa
27. US Clinical 2
28. Baiju
29. Sake
30. Mantou
31. Huangjiu
32. US Clinical 3
33. African Palm Wine
34. South American Wild
35. Chinese Wild
36. North American Wild
37. Taiwan Wild 1
38. Taiwan Wild 2
39. Asian Oak
Super clade
S1. Wine
S2. Beer
S3. Asian Fermentation
S4. Wild
Zygosity
Heterozygous
Homozygous
Ploidy
1n
2n
3n
4n
5n
