# Supplementary material for: Overview of the Saccharomyces cerevisiae population structure through the lens of 3,034 genomes
Source: G3 (Bethesda). 2024 Nov 19;14(12):jkae245. doi: 10.1093/g3journal/jkae245 (PMC11631439; doi:10.1093/g3journal/jkae245)
Supplement: jkae245_Supplementary_Data [file jkae245_supplementary_data.zip › Supplemental_Figure_Legends_G3-2024-405400.docx]

**Supplemental Figure Legends**

**Fig. S1 | Correlation between the chromosome length and the number of aneuploidies found in the 3,034 population.**

**Fig. S2 | InDels rarefaction curves**. Both all InDels (dark blue) and non-singleton InDels (light blue) were used. The curve for all InDels was fitted with a power law curve ($y=3,027\times x^{0.374}-3,377$), and the curve with non-singleton InDels with a Michaelis-Menten equation ($y=(33,450\times x)/(208+x$). The horizontal line represents the estimated total number of non-singleton InDels.

**Fig. S3 | Residual plots for the non-linear regression of the rarefaction curves. a** and **b** refer to the non-linear regression on SNPs rarefaction (Fig. 1E), with **a** corresponding to the power law regression on all SNPs and **b** the Michaelis-Menten regression on non-singleton SNPs. **c** and **d** refer to the non-linear regression on InDels rarefaction (Fig. S2), with **c** corresponding to the power law regression on all InDels and **d** the Michaelis-Menten regression on non-singleton InDels.

**Fig. S4 | Structure plot of the 3,034 population.** The structure plot is represented for the chosen value of K=38. The clade and superclade attributed to each isolate are indicated below.

**Fig. S5 |** **Neighbor-joining tree with clade, zygosity and ploidy information.** The neighbor-joining tree was built using biallelic SNPs and is represented without distance information. Isolates are colored according to the 39 clades and 4 superclades that were identified. Zygosity and ploidy information are indicated for each strain. Heterozygosity is defined here as more than 500 heterozygous SNPs per isolate.
