# Supplementary figures and images for: Overview of the Saccharomyces cerevisiae population structure through the lens of 3,034 genomes
Source: G3 (Bethesda). 2024 Nov 19;14(12):jkae245. doi: 10.1093/g3journal/jkae245 (PMC11631439; doi:10.1093/g3journal/jkae245)

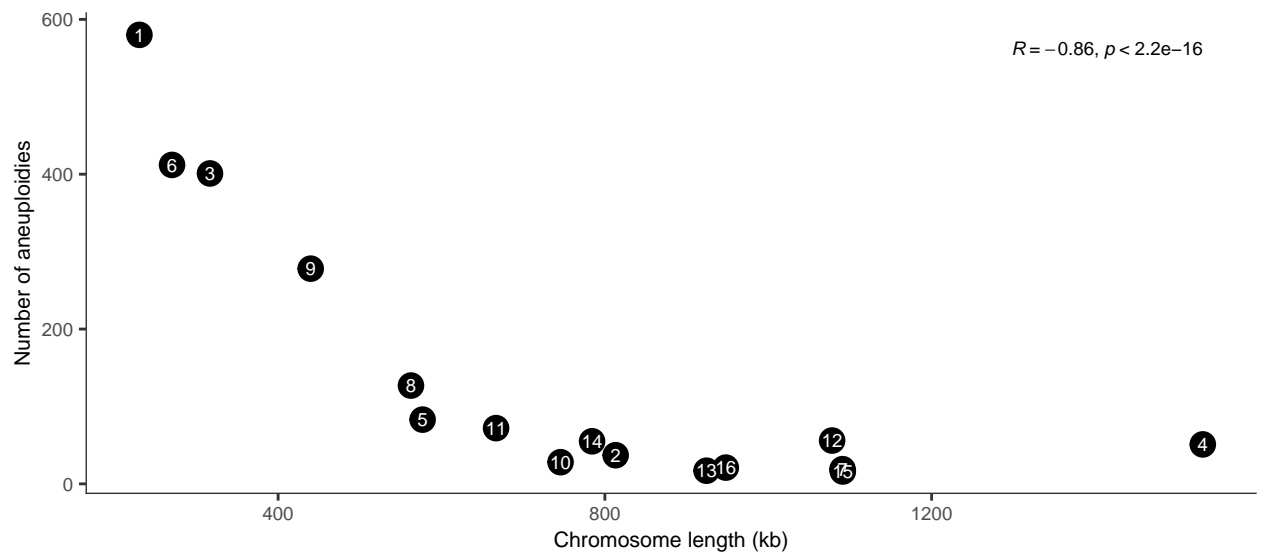

Supplement: jkae245_Supplementary_Data [file jkae245_supplementary_data.zip › Figure_S1_G3-2024-405400.pdf]

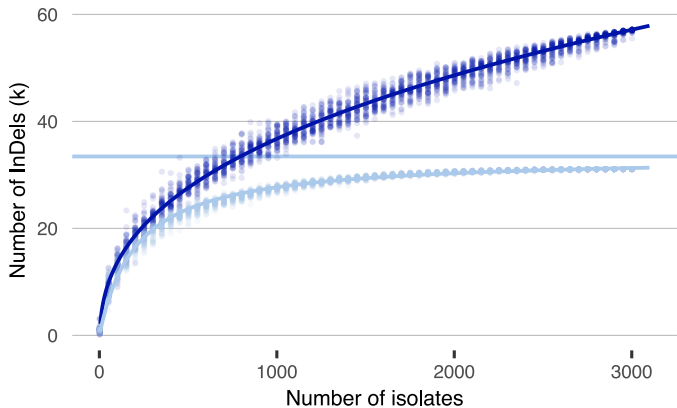

• All InDels • Non Singletons

Supplement: jkae245_Supplementary_Data [file jkae245_supplementary_data.zip › Figure_S2_G3-2024-405400.pdf]

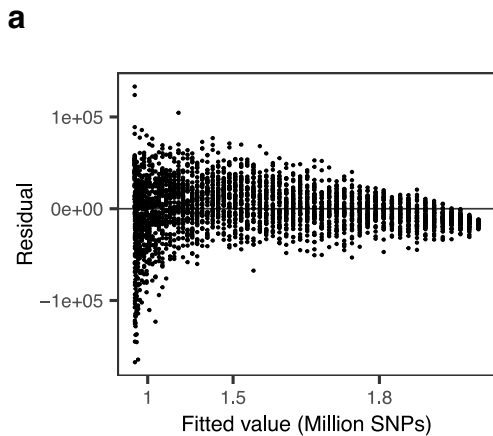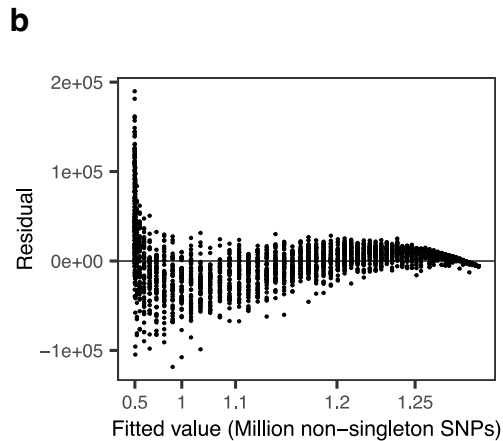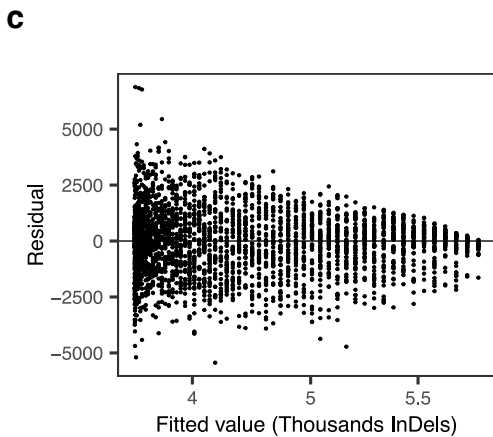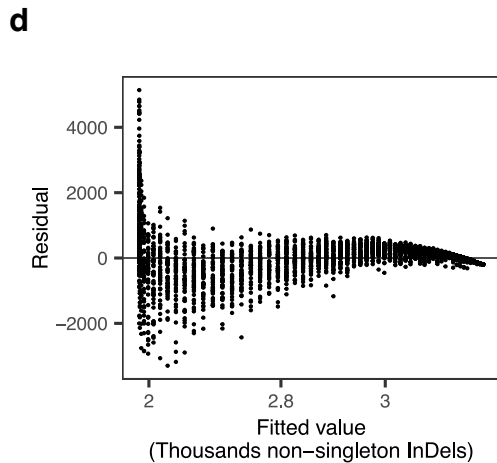

Supplement: jkae245_Supplementary_Data [file jkae245_supplementary_data.zip › Figure_S3_G3-2024-405400.pdf]

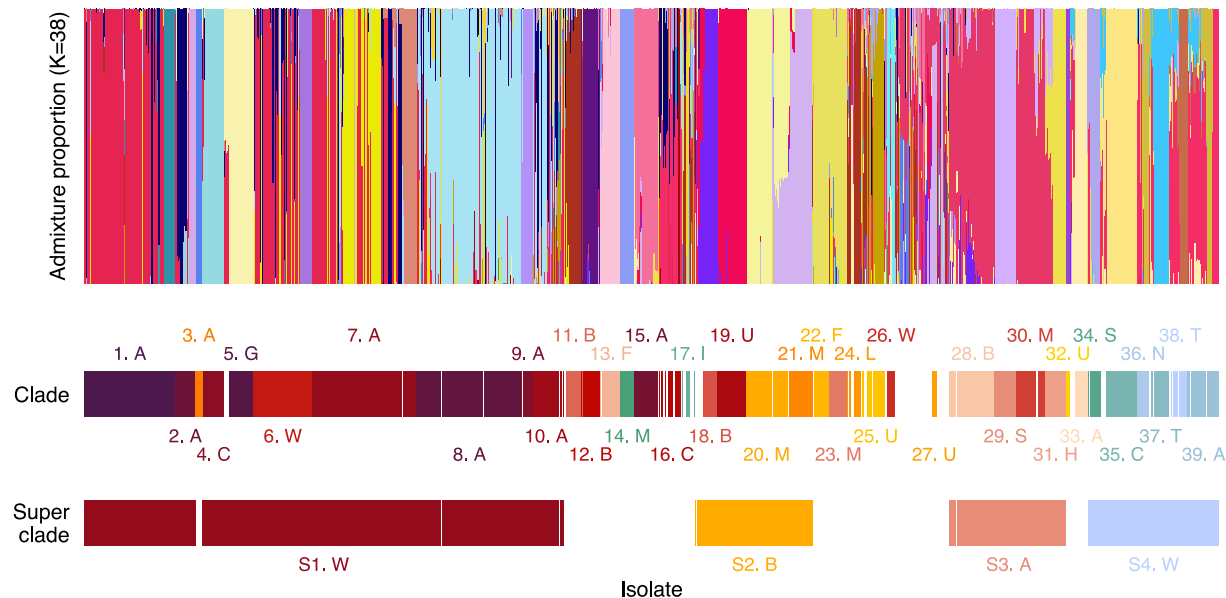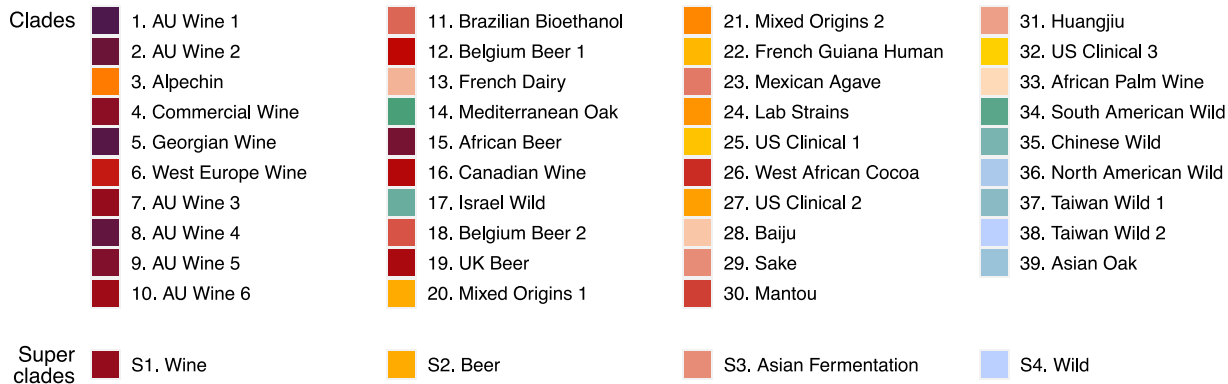

Supplement: jkae245_Supplementary_Data [file jkae245_supplementary_data.zip › Figure_S4_G3-2024-405400.pdf]
